# Prospective harmonisation of four international randomised controlled trials in Canada, China, India and South Africa: the Healthy Life Trajectories Initiative

**DOI:** 10.1136/bmjopen-2024-086233

**Published:** 2025-03-03

**Authors:** Julie Bergeron, Anouar Nechba, Samuel El Bouzaïdi Tiali, Stephanie Atkinson, Catherine Birken, Catherine Draper, Ghattu V Krishnaveni, William Fraser, Cindy Lee Dennis, Nadia Abdelouahab, Flavia Marini, Kalyanaraman Kumaran, Shane A Norris, Stephen Lye, Stephen G Matthews, He-Feng Huang, Elizabeth A Bojarski, Rayjean Hung, Jianxia Fan, Jean-Patrice Baillargeon, Isabel Fortier

**Affiliations:** 1 Research Institute of the McGill University Health Centre, Montreal, Québec, Canada; 2 McMaster University, Hamilton, Ontario, Canada; 3 University of Toronto, Toronto, Ontario, Canada; 4 MRC-Wits DPHRU, University of the Witwatersrand, Johannesburg, South Africa; 5 Holdsworth Memorial Hospital, Mysore, India; 6 Université de Sherbrooke, Sherbrooke, Quebec, Canada; 7 University of Southampton, Southampton, UK; 8 Lunenfeld-Tanenbaum Research Institute, Toronto, Ontario, Canada; 9 Shanghai Jiao Tong University, Shanghai, China

**Keywords:** Methods, Randomized Controlled Trial, Surveys and Questionnaires

## Abstract

**Abstract:**

**Objectives:**

The Healthy Life Trajectories Initiative (HeLTI) is an international multistudy consortium that supports the development and integration of four randomised controlled trials (RCTs) conducted in South Africa, India, China and Canada. HeLTI aims to evaluate interventions to improve the health and well-being of mothers and children, starting from preconception through pregnancy and early childhood until age 5 years. This paper describes the process by which we prospectively harmonised the participating studies and provides a descriptive analysis of the study-specific harmonisation potential.

**Design:**

Prospective harmonisation of four international RCTs.

**Methods:**

A list of core variables to be collected across ten waves of data collection was defined. Taking this list into consideration, investigators developed country-specific questionnaires that were then assessed and adjusted to optimise the harmonisation potential across countries. As questionnaires were not identical, where required, processing scripts were generated to help transform the collected data into the core variable format.

**Setting:**

The four RCTs are conducted in Canada, China, India and South Africa. The prospective harmonisation was led by the Maelstrom Research team in Canada.

**Participants:**

Between 4500 and 6000 women planning to get pregnant are recruited in each RCT. Women remain in the study if they become pregnant inside the planned interval of 1–3 years, depending on the country.

**Results:**

A total of 1962 variables from questionnaires, physical measurements and biospecimen analyses were defined across 10 timepoints of data collection and 3 subpopulations (mothers, partners and children). These variables cover 47 different domains of information. For the preconception phase, following the development of questionnaires and their implementation in the data collection software, 77.2% of the core variables defined can be created across the four studies.

**Conclusion:**

The HeLTI harmonisation process was successful, and the datasets generated represent a valuable resource allowing researchers to address a wide range of research questions on the impact of behaviour change interventions on maternal and child health indicators in different populations.

**STRENGTHS AND LIMITATIONS OF THIS STUDY**

- The rigorous harmonisation developed and implemented in the project led to high levels of compatibility in the data collected across the four participating studies and quality of the harmonised datasets.
- The large sample size resulting from the co-analysis of the four harmonised study datasets will allow the investigation of less frequent outcomes and interactions between risk factors.
- Comparing results across different settings and populations is made possible by prospectively harmonising data from these four studies in South Africa, India, China and Canada.
- In some cases, it was impossible to reach consensus between the four studies on the selection and definition of specific variables to include in the data collection as there were no common measures applicable to the four different settings.
- Some variables could not be generated across all four studies owing to different standardised questionnaires being used or because the wording of the questions used was deemed incompatible with the agreed-on variable definition.

## Introduction

 The Developmental Origin of Health and Diseases (DOHaD) hypothesis suggests that adversity during early life influences the risk of non-communicable disease (NCD) in adults.^1^ For example, low birth weight, poor infant nutrition and rapid childhood weight gain and obesity are well-established risk factors for the development of NCDs later in life.^2 3^ A DOHaD approach can be implemented to address the increasing burden of NCDs worldwide by providing interventions to young women to ensure they approach pregnancy in optimum health, to pregnant women to ensure a healthy pregnancy and safe delivery, and to infants and children to prevent excessive childhood adiposity and promote child development.^4 5^ Prescription for such interventions will not be universal but should be provided in the context of local food supply, healthcare programming and education level of the target population.^6^ Randomised controlled trials (RCTs) have been established to test the efficacy of various interventions, primarily during pregnancy, on maternal and child health outcomes.

The Healthy Life Trajectories Initiative (HeLTI), established in 2017, is a multi-site research collaboration to support the development of four harmonised RCTs conducted in South Africa,^7^ India,^8^ China^9^ and Canada,^10^ and supported by the WHO. Applying a DOHaD approach, HeLTI evaluates the effect of an integrated four-phase intervention starting in the preconception period and continuing through pregnancy into infancy and early childhood (up to 5 years) on reducing childhood adiposity, a known driver of NCD risk. In all sites, the integrated intervention package promotes behaviour change to optimise maternal preconception body weight, improve diet and physical activity during preconception and pregnancy, and health during pregnancy, reduce perinatal depression, increase exclusive breastfeeding, and improve parental nurturing care. Notably, the interventions are adapted to the specific context and needs of each country, involving diverse public health platforms that are nationally available for delivering the interventions.

To optimise the potential of collaborative research in the HeLTI consortium, it was essential to prospectively harmonise the information to be collected by the participating studies. Agreement beforehand on the different timepoints of data collection during the follow-up of women and their children (if they become pregnant), as well as on a core set of variables to include in each data collection was necessary to ensure that future co-analysis would be possible across the studies. Co-analysing the available individual participant data allows to obtain the statistical power required to refine investigation of the direct and interactive associations between environmental and lifestyle exposures and the interventions on the children’s adiposity and development. Moreover, it allows the pursuit of comparative research between participating countries, a key component of HeLTI in order to identify populations and settings where the interventions could be the most beneficial. This paper outlines our approach to prospectively harmonising the information to be collected through questionnaires, physical measurements and biospecimen collections. It also presents descriptive analyses of the prospective harmonisation work through assessment of study-specific questionnaires and data dictionaries, and illustrates different challenges faced throughout the process.

## Methods

### Participating studies

**Figure 1:**
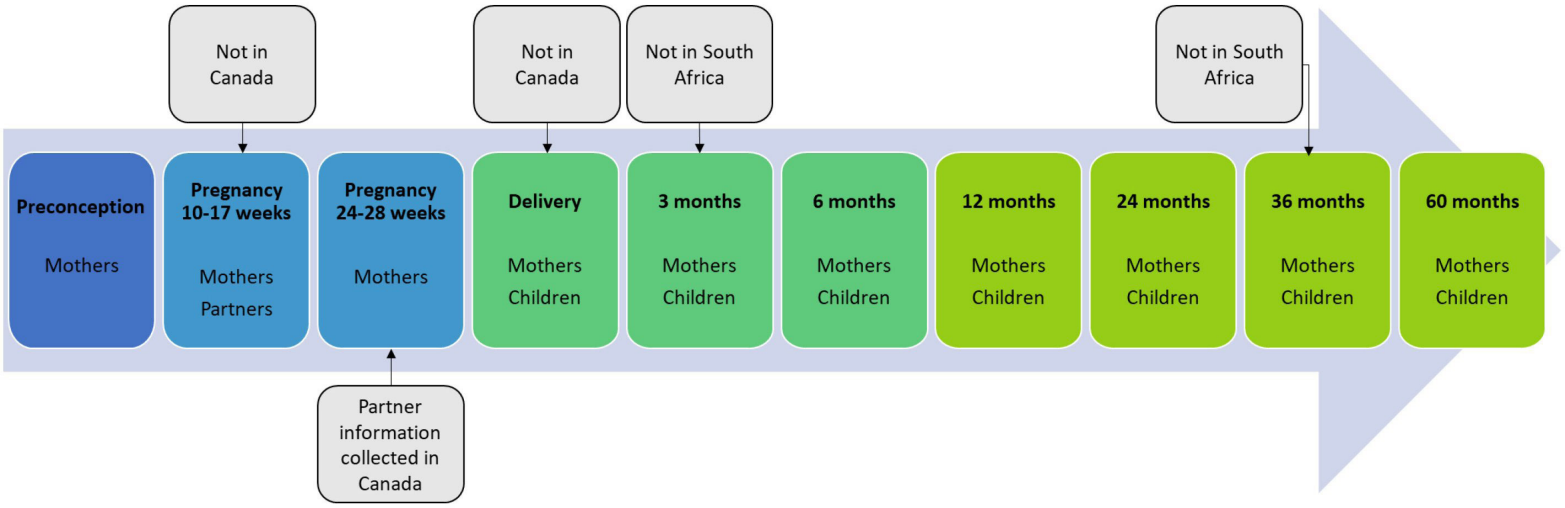
Timepoints of data collection per subpopulation.

Four RCTs were developed in the context of HeLTI in South Africa (BUilding Knowledge and a foundation for HeALthy lIfe trajectories (BUKHALI),^7^ India (Early Interventions to Support Trajectories for Healthy Life in India (EINSTEIN),^8^ China (Sino-Canadian Healthy Life Trajectories Initiative (SCHeLTI)^9^ and Canada (Trajectories of Healthy Life using Public Health and Primary Care Interventions in Canada (HeLTI Canada).^10^ Between 4500 and 6000 women planning to get pregnant in the next 1–3 years are recruited by each study, with China also recruiting some of their participants during their first trimester of pregnancy. Women are randomised to receive either standard care or the study intervention. Women who conceive during the prespecified time interval (1–3 years) are followed through pregnancy (at 10–17 weeks and 24–28 weeks of gestation) and up until their child born in the study is 5 years of age. For data collection, 10 common timepoints were agreed on, from preconception to the children reaching 5 years, with each site participating in at least 8 timepoints of data collection (figure 1). Mothers are followed-up at each of these 10 timepoints, and children seven times between delivery and 5 years of age. Partners agreeing to participate are also interviewed at the first pregnancy visit. In each subpopulation, information is collected by questionnaires, physical measures are taken and biospecimens are collected for future analyses.

### Selection and definition of core variables

To ensure compatibility of the data collected across HeLTI studies, before starting the data collection process, all study teams agreed on a common set of protocols, variables and standardised questionnaires. To succeed in this endeavour, we undertook a rigorous process based on the expertise developed by Maelstrom Research.^11^,^13^ A multidisciplinary team including data harmonisation specialists, researchers and project managers from each study was created. While the harmonisation working group (HWG) was responsible for the selection of core variables to be collected using questionnaires, the SOPs for physical measurements and biospecimen collections were established by a separate group of experts. From 2017 to 2021, the HWG held a series of online meetings to define and agree on a core set of information to be collected by participating studies, starting with the preconception data collection and continuing through each of the follow-ups. First, consensus was achieved on the topics to be covered during each data collection (eg, smoking, depression) according to the diverse HeLTI research interests. Then, a list of possible variables and variable format for each topic was defined (eg, current smoking status with ‘does not smoke’, ‘occasional smoker’ and ‘daily smoker’ as response options, and the number of cigarettes smoked per week as an integer variable), keeping in mind that they ideally had to be relevant in the four study populations. For a variable to be included, at least three out of the four studies had to retain it for data collection. Where relevant, available standardised questionnaires (eg, PHQ-9 to measure depression) were selected.

Following the discussions in the HWG, each study’s representative reviewed the proposed list of variables and standardised questionnaires with their local team to validate the decisions. During the next meeting, each representative provided feedback on their team’s assessment. Most often, more than one discussion was required before final consensus was achieved regarding the specific variables to include and their definition. If a variable was not relevant to more than one country or if it was impossible to find a common definition which could apply to the different contexts and populations, the variable was removed from the list of core variables. The same applied to the selection of standardised questionnaires, which must have been validated in the different populations and available in the appropriate languages. This work resulted in a list of core variables that could be generated by at least 3 of the participating studies.

### Development of questionnaires

**Figure 2:**
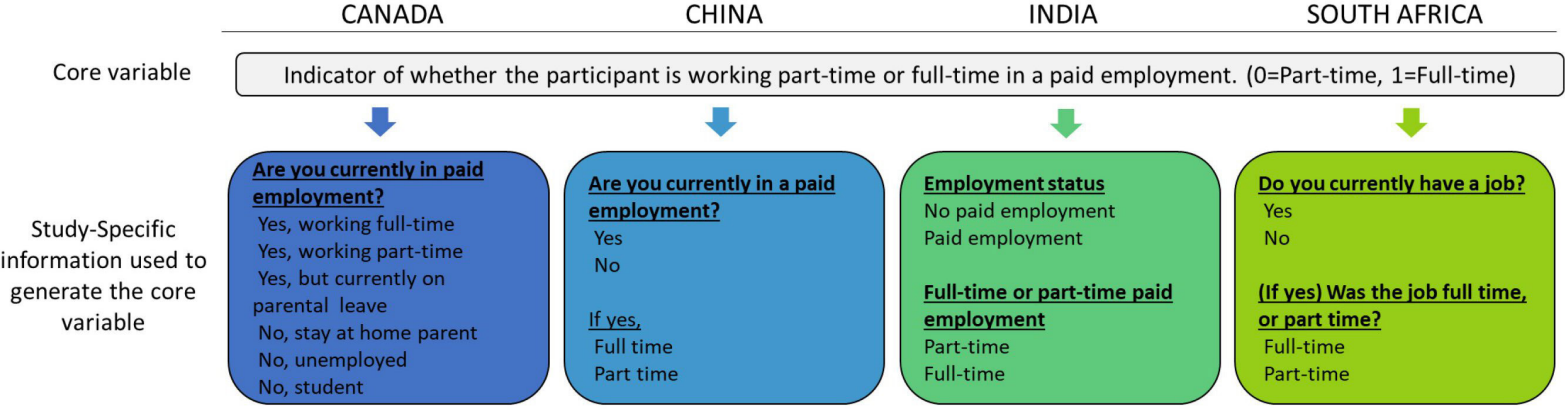
Example of the different study-specific questions allowing the generation of the core variable regarding the working schedule.

Once variable definitions were finalised for a given wave of data collection, each team created their study-specific questionnaires, in their country-specific languages. Questions included could have a different format or wording from one study to the other, as long as the meaning of the questions remained consistent with the agreed-on core variables and allowed transformation of collected data under the core variables format (example in figure 2). When a standardised questionnaire was selected, all questions had to be included without any modification from the original formulation.

### Assessment of the harmonisation potential of the draft questionnaires

Before initiating data collection, the English version of draft questionnaires created by the study teams was reviewed by Maelstrom Research’s harmonisation experts to assess their harmonisation potential with the list of core variables. Each questionnaire was carefully evaluated to identify missing questions or incompatible wording or response options that would prevent the creation of the core variables. If issues were found, they were reported to the study teams with suggested solutions. Where possible, changes were made to the questionnaires to improve the harmonisation potential. The study-specific questionnaires were then programmed in independent REDCap^14^ instances installed on local servers for data collection. While the questionnaires were programmed in different languages, the metadata describing the variables (data dictionaries) were documented in English to facilitate international collaborations.

### Assessment of the harmonisation potential of the final questionnaires and data dictionaries, and generation of processing scripts

Once the questionnaires were finalised and programmed into REDCap, data dictionaries for each timepoint of collection were extracted and reviewed by the harmonisation team to generate the final harmonisation potential. The harmonisation status was considered complete if the collected data allowed the creation of the core variable, either by being identical to the core variable or by being compatible, thus requiring the generation of a processing script to transform it under the core variable format. The harmonisation status was considered impossible if information was not collected or incompatible with the core variable format. The processing scripts were developed according to the Maelstrom standards to be compatible with Rmonize,^15^ a R package created to facilitate processing of collected data into harmonised data. As the individual participant data are accessible only to the study teams, the harmonisation scripts were sent to each team’s data manager to be run locally, with support and guidance from the Maelstrom team. All information generated through the harmonisation process (definition of variables, harmonisation potential and processing scripts) is documented and made available in accordance with the FAIR principles.^16^

### Descriptive statistics

Analyses were performed to obtain descriptive statistics presenting the results of each of the harmonisation phases (ie, selection and definition of core variables, harmonisation potential of draft questionnaires and harmonisation potential of final questionnaires and data dictionaries). For the selection and definition of the core variables, all 10 timepoints of data collection and all subpopulations (ie, mothers, partners and children) were included in the analyses. Only timepoints up until delivery were included in the assessment of the draft questionnaires’ harmonisation potential, as questionnaires were not yet available for all countries for the subsequent timepoints of data collection. Biospecimen information was excluded from the harmonisation potential as this aspect was under the purview of a separate working group. For the assessment of final questionnaires and data dictionaries, only the women’s preconception collection was included in the analyses as this was the only timepoint for which the processing scripts were finalised for all studies.

### Patient and public involvement

Neither patients nor the public were involved in this study.

## Results

### Selection and definition of core variables

Following discussions within the HWG and the biospecimen working group, 1962 core variables from questionnaires, physical measures and biospecimen analyses were defined across 10 timepoints of data collection and 47 domains of information. From those, 79.6% were planned to be collected by all four HeLTI studies. Half of the variables (50.8%) were taken from standardised questionnaires and the other half was composed of variables for which definitions were provided, but no exact question wording was imposed. In some cases, topics initially of interest did not lend themselves to prospective harmonisation since there were no common measures appropriate for the different contexts of the four countries (eg, socioeconomic status, nutrition). For these domains, retrospective harmonisation could be explored to generate common data items.^11^

**Figure 3:**
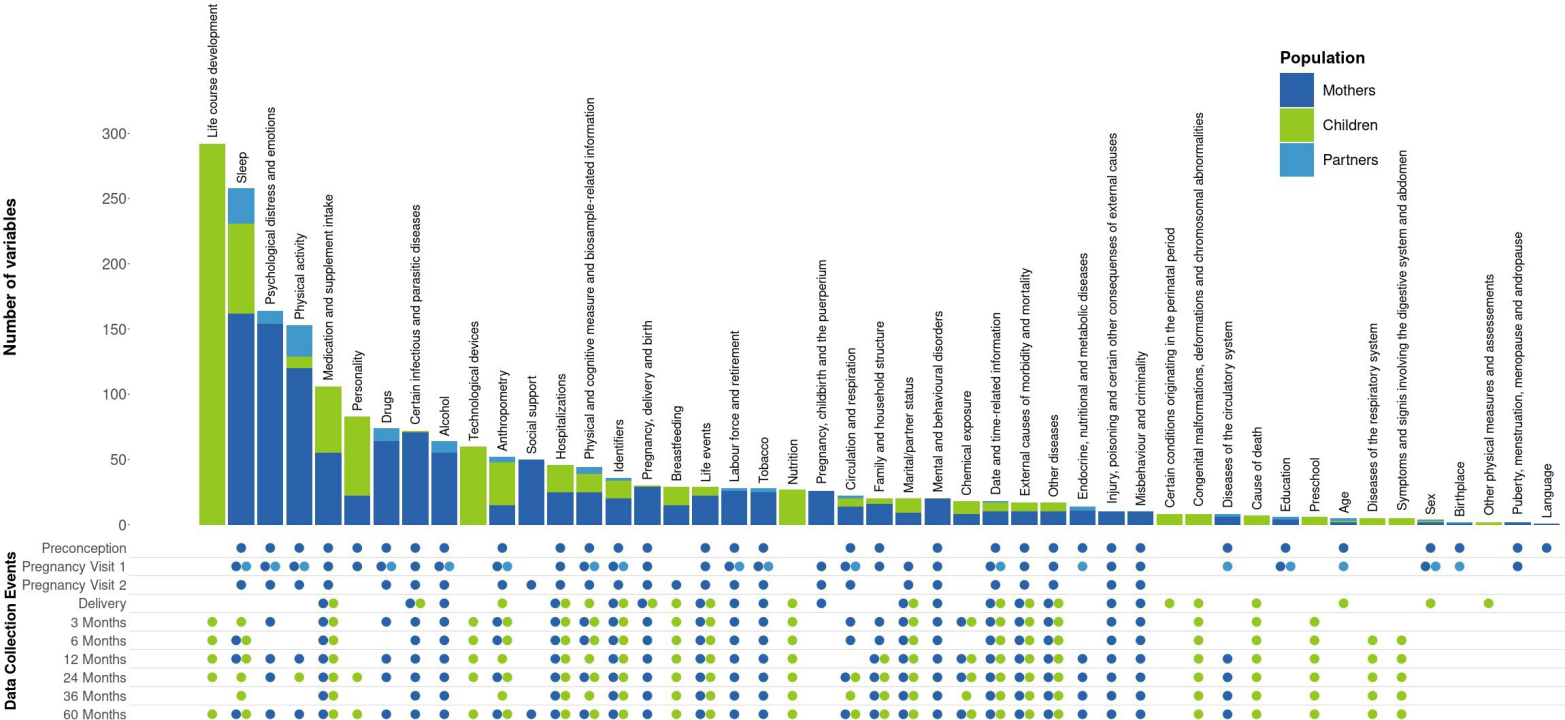
Number of variables defined per domain of information for each subpopulation and their timepoints of data collection.

For mothers, 1049 variables were agreed on across the ten waves of data collection, covering 39 domains of information (figure 3). The domains with the most variables collected are sleep, psychological distress and emotions, and physical activity. For partners, only one wave of data collection was prospectively harmonised, and 110 core variables defined, with sleep and physical activity being the domains with the highest number of core variables. Finally, 7 timepoints were included in the harmonisation effort for children, for a total of 803 variables. Domains with the highest number of variables for children pertain to life-course development and sleep. A full description of the HeLTI harmonisation initiative and core variables can be found on the Maelstrom Research website (https://maelstrom-research.org/study/helti-hi). For more information about the content documented on the website and how to find it, see online supplemental material 1.

### Harmonisation potential of questionnaires and data dictionaries

**Figure 4:**
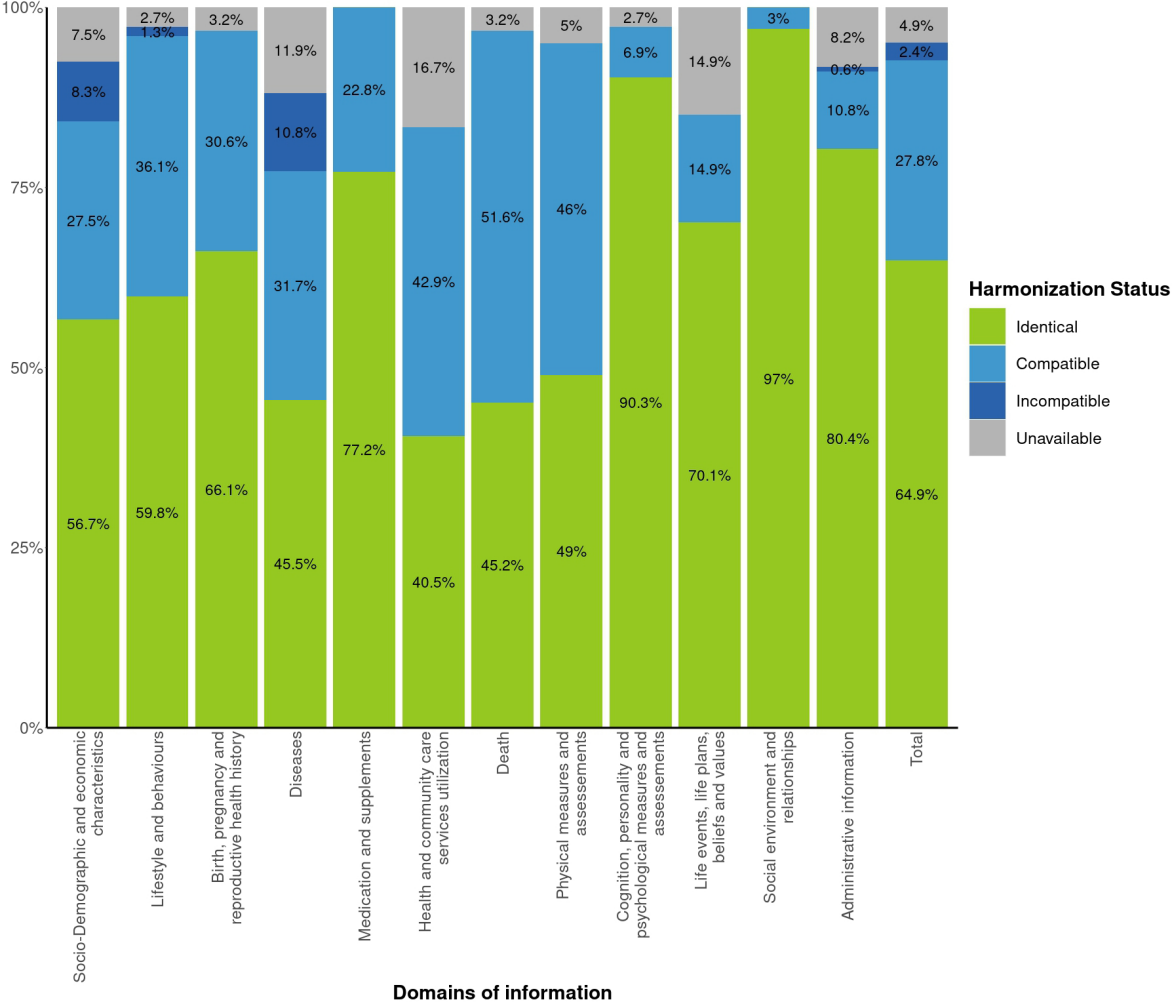
Harmonisation potential of the four HeLTI studies per domain of information, from preconception to delivery.

Figure 4 shows the evaluation of the harmonisation potential per domain of information using the maternal and partner study-specific draft questionnaires for four of the data collection timepoints (preconception, both pregnancy visits and delivery). According to this evaluation, 64.9% of the study-specific questions were in an identical format (wording and categories) to the core variables. Another 27.8% of the study-specific questions were compatible with the core variable format, but required data transformation. The harmonisation results varied across domains of information, with some having a higher percentage of variables being possible to generate (eg, social environment and relationships) than others (eg, diseases). The use of standardised questionnaires also led some domains to have a higher percentage of identical variables. In the end, according to the draft questionnaires, 92.7% of the study-specific harmonised variables could be generated, with 79.5% of the core variables from preconception to delivery being harmonisable across all four participating studies.

**Figure 5:**
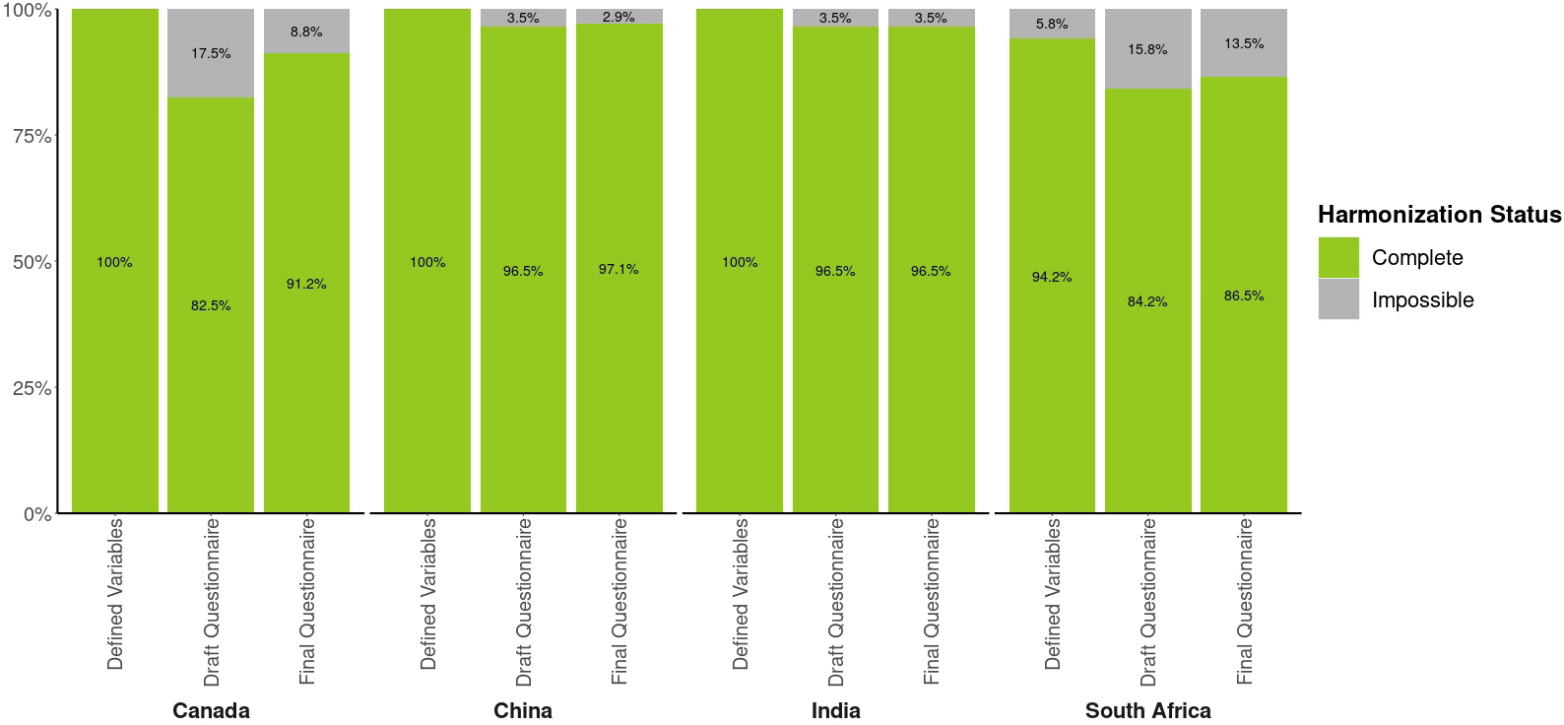
Harmonisation potential of the preconception data collection for each step of the process.

When compared with the results from the selection and definition of core variables, we observed a diminution in the percentage of harmonisable variables in the preconception draft questionnaire for all four studies (figure 5). This varied from a 3.5%–17.5% decrease in core variable compatibility owing to information being left out or to changes made to the variable definitions during the development of questionnaires. Taking into consideration the feedback provided following the assessment of the draft questionnaires, the study teams were able to make corrections and slightly improve the compatibility of the information collected with their final version before implementation into REDCap. Thus, depending on the country, 86.5%–97.1% of preconception core variables were possible to generate according to their final questionnaires and data dictionaries. However, some collected information remained incompatible with the agreed format or was purposely removed from the list of data to collect. Ultimately, 77.2% of the agreed-on preconception core variables could be generated across the four countries and thus are available for future co-analysis including all participating studies. The core variables impossible to generate across the four studies are dispersed across various domains of information such as adverse events, COVID-19, and alcohol and tobacco consumption. In most cases, the information was simply not collected by one of the studies. For example, in South Africa, a different questionnaire was used to collect information about alcohol consumption, thus it was impossible to generate all core variables about alcohol for this country. In less prevalent cases, like for COVID-19, information was collected, but some questions asked were incompatible with the core variable definition. This means that most variables about COVID-19 were generated across the four studies, with a few exceptions. Assessment of the harmonisation potential of the following data collection timepoints is ongoing.

### Generation of processing scripts

Using the final questionnaires and data dictionaries extracted from REDCap, processing scripts to transform the preconception collected data under the core variable format were developed, and harmonised datasets generated. An example of the processing script to harmonise the information collected by the studies about the mothers working part-time or full-time during preconception is provided in online supplemental figure 1. This work is also ongoing for the following timepoints of data collection.

## Discussion

### Principal findings

The harmonisation process performed in HeLTI was successful, and consensus could be reached on a large amount of information to be collected by the four participating studies. Through the work of the HWG, over 1900 core variables were defined and selected. Following the study-specific questionnaires’ creation and programming into the data collection software, 77.2% of the preconception variables remained harmonisable across the four countries, and thus available for future co-analyses to address a wide range of research questions.

### Strengths and limitations

HeLTI aims to generate harmonised data from four studies in South Africa, India, China and Canada, which when co-analysed, will result in a large sample size allowing the examination of less frequent outcomes and the exploration of interactions between risk factors. The diversity of settings in the different countries also allows the comparison of results across various populations, especially regarding the impacts of the interventions. Another strength of the initiative is the international and multidisciplinary collaboration. The breadth of expertise coming from the contrasting national contexts guaranteed the selection of the most appropriate information to collect for the co-analyses. The rigorous harmonisation approach developed and implemented across the lifespan of the project was critical in achieving the high level of compatibility in information collected across countries and leads to producing high-quality harmonised datasets. The early investment in this process will also reduce the effort and resources needed for data cleaning and preparation for analysis. Additionally, HeLTI was developed to serve as a data platform to be used by the international scientific community. Thus, comprehensive documentation is provided to users including: the specific design of each participating study and the variables they collected, the list of harmonised variables, their definition and the harmonisation potential across countries.^17^ Having the questionnaires and variable definitions available on the website could also leverage harmonisation with future studies, as they could use the material to get insight for their own questionnaires.

However, some difficulties were encountered in the process, resulting in limitations to the generated datasets. Agreement on the standardised questionnaires to use was challenging as many were not available in all languages or validated for all country-specific populations. While we consistently aimed to reach consensus, in some cases, teams had to select a different questionnaire or a different version of a questionnaire to suit the local context. In these cases, the variables collected were deemed incompatible with those of the other countries, leading to a diminution in harmonisation potential. Also, reaching consensus on the core variable definitions was difficult, as each team needed to ensure questions were optimal to collect information locally, while defining generic variables that could be applied to all settings. Moreover, during the questionnaire creation phase, some questions were worded in a way that was incompatible with the agreed-on core variable definitions. While feedback was provided to modify those questions, sometimes data collection had already started, and the questionnaires could not be changed, or the team preferred not to modify the question. Again, this led to some core variables not being generated across all studies. Finally, some topics were impossible to prospectively harmonise as there was no common measure across the four countries. For example, socioeconomic status is assessed very differently in rural India than in Canada, thus no variable could be defined that would fit all contexts. While those variables were omitted from the prospective harmonisation, more in-depth exploration of the data collected could allow some harmonisation retrospectively. Although the issue of core variables that are impossible to generate might seem an obstacle to investigate some research questions, various solutions can be considered. Researchers might decide to perform analyses using data from only three of the four countries. They could also choose to use a different harmonised variable in the same domain of information or generate a new one using a more inclusive variable definition.

### Interpretation

The large harmonised dataset generated in HeLTI is an invaluable resource for research on a wide array of exposures starting from preconception to pregnancy and early childhood, on key maternal and child health outcomes. Many initiatives in maternal and child research have retrospectively harmonised data already collected by studies, such as the LifeCycle project^18^ and the ReACH initiative,^19^ but prospective harmonisation of information is less frequent. While retrospective harmonisation remains a great avenue to maximise usage of available data, the harmonisation potential across studies is generally limited owing to differences in scientific objectives and in measures or questionnaires used to collect the information. In prospective harmonisation, as the researchers agree beforehand on a set of data to be collected, the harmonisation potential is substantively increased, resulting in more variables of higher quality being available for co-analysis. Thus, even though there were many challenges and some core variables could not be generated, the harmonisation potential in HeLTI remains excellent compared with past initiatives.^20^,^23^ Lastly, this paper describing the process and methods developed for the HeLTI initiative could serve as a guide for future initiatives aiming to obtain a high degree of harmonisation potential across participating studies.

## Conclusion

The harmonised datasets generated through HeLTI are an important resource for DOHaD research, as they allow investigation of various research questions across different settings, as well as determining the efficacy of interventions aiming to promote healthy behaviours and optimise maternal and child mental and physical health. Once finalised, this resource will also be available to researchers outside of the HeLTI initiative, with an access request process through each individual study.

## supplementary material

10.1136/bmjopen-2024-086233 online supplemental file 1

10.1136/bmjopen-2024-086233 online supplemental file 2

## Data Availability

Data are available upon reasonable request.
