# Supplementary material for: Prospective harmonisation of four international randomised controlled trials in Canada, China, India and South Africa: the Healthy Life Trajectories Initiative
Source: BMJ Open. 2025 Mar 3;15(3):e086233. doi: 10.1136/bmjopen-2024-086233 (PMC11877250; doi:10.1136/bmjopen-2024-086233)
Supplement: online supplemental file 1 [file bmjopen-15-3-s001.docx]

**Supplementary material 1**

**Documentation of the harmonization process and results**

This supplementary material provides an overview of the documentation of the harmonization process, core variables and harmonization results available on the Maelstrom Research website.

**HeLTI network**

<https://maelstrom-research.org/network/helti>

The network description includes information about the main objectives of the Healthy Life Trajectories Initiative, the list of investigators leading the initiative and a link to the initiative’s website. This page also includes a list of the four participating randomized clinical trials (RCT).

**HeLTI participating studies**

South Africa: <https://maelstrom-research.org/study/bukhali>

India: <https://maelstrom-research.org/study/einstein>

Canada: <https://maelstrom-research.org/study/helti-canada>

China: <https://maelstrom-research.org/study/schelti-life-tree>

Each of the four RCTs part of HeLTI is also described independently. The study description includes information about the study-specific objectives, investigators, design, sub-populations of mothers, partners and children (number of participants, selection criteria and sources of recruitment) and the timing and content of each data collection for each sub-population. A full list of variables collected at each timepoint of data collection is also documented for each study. These variables include the ones that were prospectively harmonized, but also additional variables that are specific to each country.

**HeLTI harmonization initiative**

<https://maelstrom-research.org/study/helti-hi>

The description of the harmonization initiative provides information about the objectives specific to the harmonization process, the person to contact regarding the harmonization work, the list of participating studies and the list of harmonization protocols (see below). In the case of HeLTI, there is one protocol listed per sub-population and timepoint of data collection. This page also includes a figure presenting the different domains of information covered by the variables prospectively harmonized across all data collections.

**HeLTI harmonization protocols**

e.g., <https://maelstrom-research.org/dataset/helti-m-pc-hp>, others are listed on, and accessible through the harmonization initiative page.

Each planned data collection has an associated harmonization protocol detailing the type of harmonization, the approach, procedures, data infrastructure in place and an overview of the variable content for the specific timepoint of collection. This page also includes a table displaying the harmonization results, including a list of the core variables selected for this data collection and the harmonization status (complete or impossible), indicating whether the data collected by each participating study allowed the generation of the core harmonized variable. More information on the core variables is available by cliquing on the variable name in this table, including their label, description, response options for categorical variables and classification by areas of information.
