# Supplementary figures and images for: Prospective harmonisation of four international randomised controlled trials in Canada, China, India and South Africa: the Healthy Life Trajectories Initiative
Source: BMJ Open. 2025 Mar 3;15(3):e086233. doi: 10.1136/bmjopen-2024-086233 (PMC11877250; doi:10.1136/bmjopen-2024-086233)

Supplemental Figure 1: Example of the processing scripts used to generate the core variables

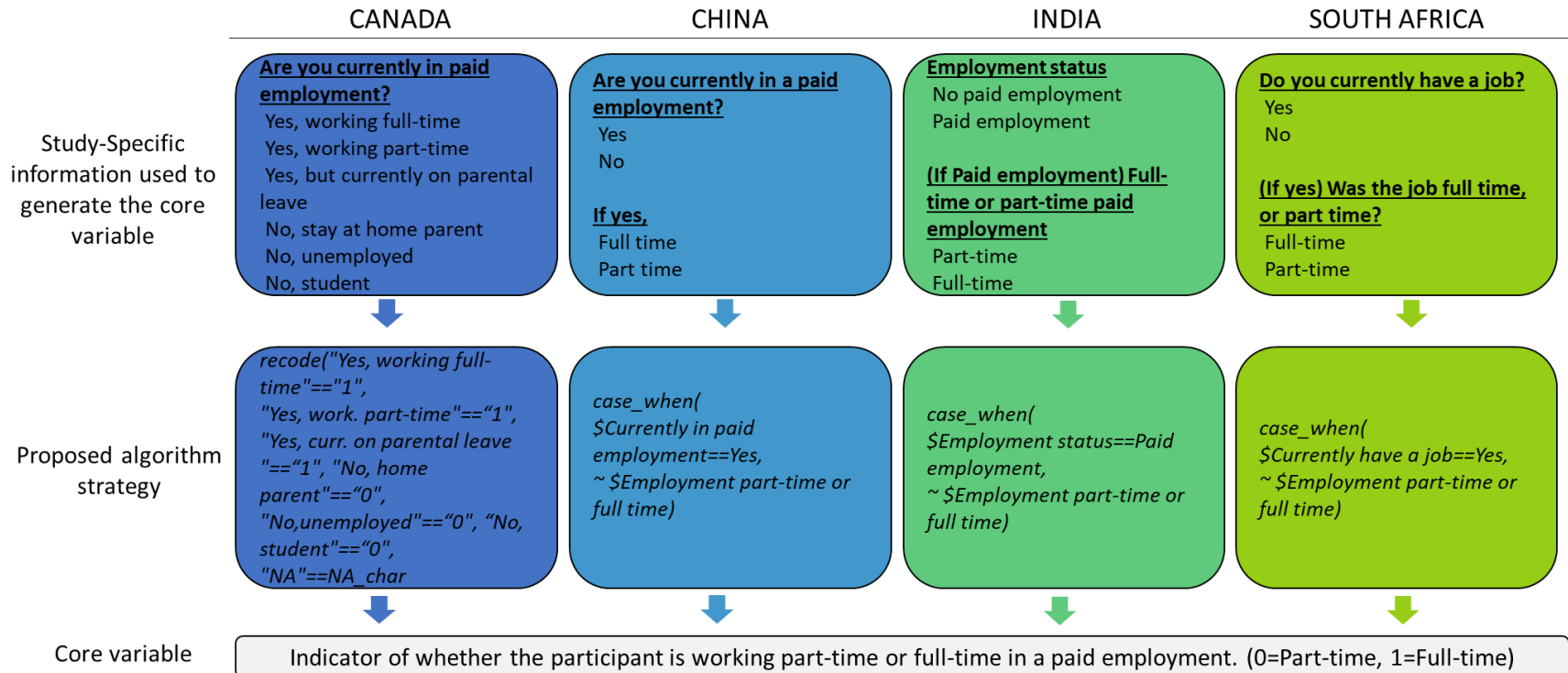

Supplement: online supplemental file 2 [file bmjopen-15-3-s002.pdf]
