# Supplementary material for: Prospective harmonisation of four international randomised controlled trials in Canada, China, India and South Africa: the Healthy Life Trajectories Initiative
Source: BMJ Open. 2025 Mar 3;15(3):e086233. doi: 10.1136/bmjopen-2024-086233 (PMC11877250; doi:10.1136/bmjopen-2024-086233)
Supplement: Supplementary file 3 [file bmjopen-2024-086233.reviewer_comments.pdf]

PEER REVIEW HISTORY

BMJ Open publishes all reviews undertaken for accepted manuscripts. Reviewers are asked to complete a checklist review form and are provided with free text boxes to elaborate on their assessment. These free text comments are reproduced below.

ARTICLE DETAILS

Title (Provisional)

Prospective harmonization of four international randomized controlled trials in Canada, China, India and South Africa: The Healthy Life Trajectories Initiative (HeLTI)

Authors

Bergeron, Julie; Nechba, Anouar; El Bouzaïdi Tiali, Samuel; Atkinson, S; Birken , Catherine; Draper, Catherine; Krishnaveni, Ghattu V; Fraser, William; Dennis, CindyLee; Abdelouahab, Nadia; Marini, Flavia; Kumaran, Kalyanaraman; Norris, Shane A; Lye, Stephen; Matthews, Stephen G; Huang, He-Feng; Bojarski, Elizabeth A.; Hung, Rayjean; Fan, Jianxia; Baillargeon, Jean-Patrice; Fortier, Isabel

---

VERSION 1 - REVIEW

**Reviewer**	**1**
**Name**	**Rinaldi, Eugenia**
**Affiliation**	**Charité Universitätsmedizin Berlin**
**Date**	**29-May-2024**
**COI**	**I have no competing interests**

Interesting example of harmonization of studies in a relevant field. However, I believe more details on the core data set agreed (how many variables? which information categories?) or maybe even an xls file as suplementary material would help re-produce the work. If this is available on a website please give details o how to access it. Some other harmonization initiatives are mentioned but it would be interesting have more detail on why this approach is better. Are there other harmonization approaches? Is this approach valuable for new initiatives? If so, how?

**Reviewer**	**2**
**Name**	**Thomas, Roger**
**Affiliation**	**University of Calgary, Family Medicine**
**Date**	**11-Jul-2024**

**COI**	**none**

This is a complicated and meritorious task.

This is a straightforward description of your harmonisation process.

The article would have added value if you could please address these questions:

A key conclusion is: "Thus, depending on the country, 86.5% to 97.1% of preconception core variables are possible to generate according to their data dictionaries. However, some collected information remained incompatible with the agreed format or was purposely removed from the list of data to collect. Ultimately, 77.2% of the agreed upon preconception core variables can be generated across the four countries and thus are available for future co-analysis including all participating studies. Assessment of the harmonization potential of the following data collection timepoints is ongoing."

[Please comment on how the 22.8% of preconception core variables which could not be harmonised will affect the ability to test which key research questions of the studies to be tested]

Figure 3: Number of variables defined per domain of information for each sub-population and their timepoints of data collection

[This is a complicated table. Please summarise how the missing data will affect the ability to test which key research questions of the studies

Also please comment for Figure 4 : Harmonization potential of the four HeLTI studies per domain of information, from preconception to delivery how this affect the ability to test your research questions.

Have your identified RCTs in the literature which will enable you to draw conclusions for the topics for which your data cannot be harmonised?

**Reviewer**	**3**
**Name**	**Chowdhury, Ranadip**
**Affiliation**	**Centre for Health Research and Development, Society for Applied Studies**
**Date**	**22-Dec-2024**
**COI**	**None**

This manuscript represents a significant contribution to the field of data harmonization. The draft is well-composed; however, I have a few technical suggestions to enhance its clarity and depth:

It would be beneficial to specify the gestational age at which pregnancy data are collected to contextualize the timing of data capture within the pregnancy timeline.

The manuscript should clearly define the term "preconception period" used within the study to ensure consistency and clarity in its application.

I recommend that the authors expand on Figure 3 by detailing the broader themes of data variables, providing a clearer overview of the data structure and categories used.

The development of a comprehensive framework that integrates epidemiological, clinical, and biochemical data would greatly enhance the utility and applicability of the harmonized dataset.

The discussion currently omits a crucial aspect: the potential use of harmonized data beyond the consortium. It is essential to address how external researchers can access and utilize this data, ensuring the manuscript's relevance to a broader scientific community.

VERSION 1 - AUTHOR RESPONSE

Reviewer: 1
Dr. Eugenia Rinaldi, Charité Universitätsmedizin Berlin
Comments to the Author:
Interesting example of harmonization of studies in a relevant field. However, I believe more details on the core data set agreed (how many variables? which information categories?) or maybe even an xls file as suplementary material would help re-produce the work. If this is available on a website please give details o how to access it.
The detailed documentation of the harmonized datasets and variables, including information categories, is available on the Maelstrom Research website. A new supplementary material was created to explain the different components of the documentation and where to find them on the website, with links included. We hope this will help the readers browse the website to find the additional, more detailed information, on our harmonization work.

Some other harmonization initiatives are mentioned but it would be interesting have more detail on why this approach is better. Are there other harmonization approaches? Is this approach valuable for new initiatives? If so, how?
In the discussion, in the paragraph explaining the strengths of a prospective harmonization approach compared to the retrospective approach, we added information to clarify the limitations of retrospective harmonization. We also added a sentence stating the value of this paper as a guide for future initiatives.

Reviewer: 2
Prof. Roger Thomas, University of Calgary
Comments to the Author:
This is a complicated and meritorious task.

This is a straightforward description of your harmonisation process.

The article would have added value if you could please address these questions:

A key conclusion is: "Thus, depending on the country, 86.5% to 97.1% of preconception core variables are possible to generate according to their data dictionaries. However, some collected information remained incompatible with the agreed format or was purposely removed from the list of data to collect. Ultimately, 77.2% of the agreed upon preconception core variables can be generated across the four countries and thus are available for future co-analysis including all participating studies. Assessment of the harmonization potential of the following data collection timepoints is ongoing."

[Please comment on how the 22.8% of preconception core variables which could not be harmonised will affect the ability to test which key research questions of the studies to be tested]
Additional information was added to the results and discussion sections to explain how the variables impossible to harmonized could impact the research questions that can be investigated. We also provide potential solutions to this issue that could be considered by researchers interested in using the HeLTI data.

Figure 3: Number of variables defined per domain of information for each sub-population and their timepoints of data collection

[This is a complicated table. Please summarise how the missing data will affect the ability to test which key research questions of the studies
More information about the core variables and when they are collected (Figure 3) can be found on the Maelstrom Research website. A new supplementary material was created to explain how to find it on the website.
Please refer to the previous comment's response regarding changes made in the manuscript to add more information about how variables impossible to generate will affect the research questions that can be investigated.

Also please comment for Figure 4 : Harmonization potential of the four HeLTI studies per domain of information, from preconception to delivery how this affect the ability to test your research questions.
Please see answer to the first comment.
We also revised the wording used to better distinguished between the different steps of the process (selection and definition or core variables, assessment of the harmonization potential of the draft questionnaires and assessment of the harmonization potential of the final questionnaires and data dictionaries).

Have your identified RCTs in the literature which will enable you to draw conclusions for the topics for which your data cannot be harmonised?
No, because the data impossible to harmonize is spread across multiple domains of information and it would be difficult to identified new RCTs which specialised in all of them.

Reviewer: 3
Dr. Ranadip Chowdhury, Centre for Health Research and Development, Society for Applied Studies
Comments to the Author:
This manuscript represents a significant contribution to the field of data harmonization. The draft is well-composed; however, I have a few technical suggestions to enhance its clarity and depth:

It would be beneficial to specify the gestational age at which pregnancy data are collected to contextualize the timing of data capture within the pregnancy timeline.

Mention of the exact weeks of gestation for the two pregnancy visits was added to the methods section in the paragraph about participating studies. Figure 1 was also updated to include the specific weeks of pregnancy instead of the titles of visit 1 and 2.

The manuscript should clearly define the term "preconception period" used within the study to ensure consistency and clarity in its application.
In the methods section, in the paragraph about participating studies, the text was modified to make the timing of the preconception/recruitment data collection more explicit.

I recommend that the authors expand on Figure 3 by detailing the broader themes of data variables, providing a clearer overview of the data structure and categories used.
The harmonization initiative and core harmonized variables are described in details on the Maelstrom Research website. A new supplementary material was created to guide the readers navigating the website and helping them finding the additional information they are looking for.

The development of a comprehensive framework that integrates epidemiological, clinical, and biochemical data would greatly enhance the utility and applicability of the harmonized dataset.
The framework can be found in the figure on the harmonization initiative page on the Maelstrom Research website. The link to this page is provided in the new supplementary material.

The discussion currently omits a crucial aspect: the potential use of harmonized data beyond the consortium. It is essential to address how external researchers can access and utilize this data, ensuring the manuscript's relevance to a broader scientific community.
The HeLTI data will be available to external users once the data collection and harmonization is completed. To request data, users will need to contact each individual study as stated in the data availability statement. To clarify this for the reader, we added this information in the conclusion section.
